# Our Journal

**Published:** 1856-04

**Authors:** 


### OUR JOURNAL.

We would take this occasion to say that there has been much complaint recently, on the part of our subscribers, because of the non-receipt of the Journal. This has been so often repeated as to become a source of deep mortification on our part, at the present deranged condition of the mail service.

We have mailed to each subscriber, and as legibly as it could well be penned, directed, and forwarded, and if they have failed to reach each one to whom directed, it assuredly has not been our fault.

While on this subject we would remark that it is very encouraging to observe the disposition on the part of the profession of the West, to sustain this enterprise, and, also, to receive so many proofs of their approval of the matter and manner of its conduct. We shall strive not only to make it acceptable but interesting and profitable also.

The medical men of the State are assured that we do not labor to make it the organ of the institution, so much as that of the profession. In this light, we are glad to perceive it is considered. A letter before us, containing an enclosure in payment of subscription, from one of our friends, a citizen of a flourishing town in the interior, when speaking of it, uses the term "*our.*" We thank him for that word, as well as his generous promptness and consideration, and this is not the only example of the kind. Such men know the labors, responsibilities and sacrifices incident to such an undertaking, and they know how to appreciate the value of a work of their own. To labor with and for such men is a pleasure, a satisfaction, and for such we are contented to strive in the future.

We, in conclusion, can assure our friends that this work is now no longer an experiment, but a "fixed fact," as we are receiving subscribers every day, each one of whom, we trust, will consider himself a committee of "one" to solicit others to follow his example.

We would be glad to be advised always of any failure in its reception. All business matters to be addressed to Prof. J. C. Hughes. Communications for the Journal to the Editors.

